# Severe aortic stenosis has blunted myocardial T1 relaxation response to vasodilator stress: a cardiac magnetic resonance adenosine stress test study

**DOI:** 10.1186/1532-429X-17-S1-O28

**Published:** 2015-02-03

**Authors:** Masliza Mahmod, Stefan K Piechnik, Eylem Levelt, Vanessa M Ferreira, Jane M Francis, Andrew Lewis, Nikhil Pal, Houman Ashrafian, Stefan Neubauer, Theodoros D Karamitsos

**Affiliations:** 1 Division of Cardivascular Medicine, Radcliffe Department of Medicine, Oxford Centre for Clinical Magnetic Resonance Research, Oxford, UK; 2 1st Department of Cardiology, AHEPA Hospital, Aristotle University of Thessaloniki, Thessaloniki, 54636, Greece

## Background

Aortic stenosis is characterized by impaired myocardial perfusion reserve due to coronary microvascular dysfunction. T1 mapping is sensitive to myocardial water content of both intra- and extracellular in origin, but the effect of intravascular compartment changes on T1 has never been assessed previously. We aimed to assess the effect of adenosine-induced vasodilatation on native (pre-contrast) T1 values in patients with severe aortic stenosis (AS) before and after aortic valve replacement (AVR).

## Methods

42 subjects (26 patients with severe AS without obstructive coronary artery disease and 16 controls) underwent cardiovascular magnetic resonance at 3T for native T1-mapping (ShMOLLI), first-pass perfusion (myocardial perfusion reserve index-MPRI) at rest and during adenosine stress, and late gadolinium enhancement (LGE).

## Results

**Figure 1:**
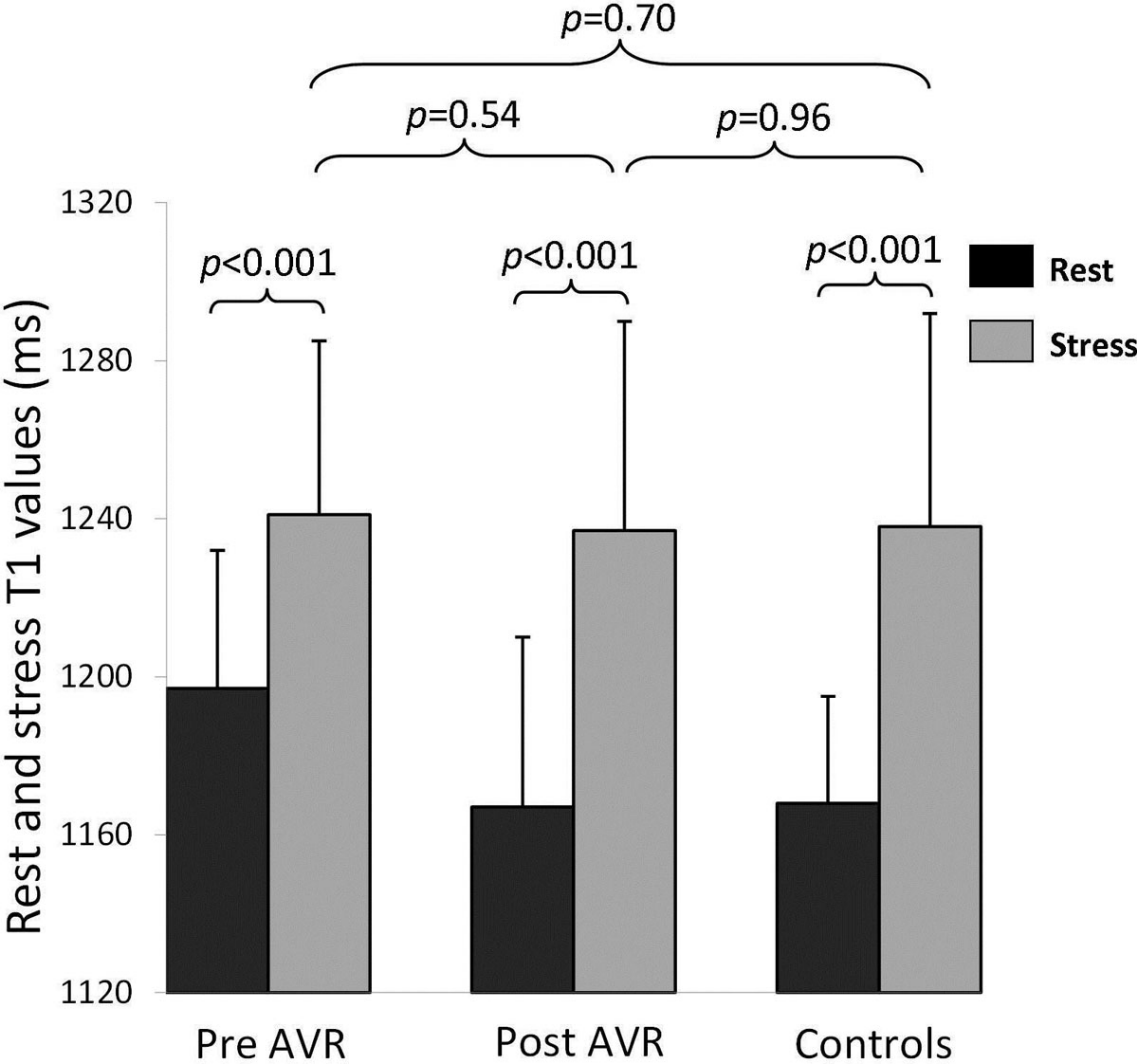
Rest and adenosine stress T1 values.

AS patients had increased resting myocardial T1 (1196±47ms vs. 1168±27ms, p=0.037), reduced MPRI (0.92±0.31 vs. 1.74±0.32, p<0.001), and increased left ventricular mass index (LVMI) and LGE volume compared to controls (Table 1). T1 values had positive correlation with LVMI, LGE volume but an inverse correlation with aortic valve area and MPRI. During adenosine stress, the maximal T1 in AS was similar to controls (1240±51ms vs. 1238±54ms, p=0.88), possibly reflecting a similar level of maximal coronary vasodilatation in both groups (Figure 1). Interestingly, the T1 response to stress was blunted in AS (ΔT1 3.7±2.7% vs. 6.0±4.2% in controls, p=0.013). Seven months after AVR (n=16) myocardial T1 and response to adenosine stress recovered towards normal.

**Table 1:** Baseline clinical characteristics of severe AS patients and normal controls

	Severe Aortic Stenosis (n = 26)	Normal controls (n = 16)	P value
Age (years)	67.8 ± 9	63.3 ± 3.4	0.06

Male, n (%)	19 (73)	8 (53)	0.16

Body mass index (kg/m2)	27.8 ± 4.5	27.0 ± 3.8	0.38

Systolic blood pressure (mmHg)	134.4 ± 18.1	131.0 ± 11.0	0.51

Diastolic blood pressure (mmHg)	74.4 ± 9.4	76.5 ± 10.2	0.51

Heart rate (bpm)	66.1 ± 9.4	64.3 ± 10.5	0.58

Peak AV gradient (mmHg)	83.1 ± 14.6	-	

CMR findings			

Aortic valve area (cm2)	0.82 ± 0.02	4.04 ± 0.75	<0.001

LV end-diastolic volume (ml)	143.2 ± 44.4	133.7 ± 33.1	0.47

LV ejection fraction (%)	74.5 ± 5.8	68.8 ± 6.4	0.005

LV mass index (g/m2)	96.0 ± 31.2	55.8 ± 13.9	<0.001

Presence of LGE, n (%)	21 (81)	0	<0.001

Myocardial T1 (ms)	1196 ± 47	1168 ± 27	0.037

Values are mean ± SD or percentages. CMR indicates cardiovascular magnetic resonance; LGE, late gadolinium enhancement; LV, left ventricular.

## Conclusions

Our findings suggest that intravascular compartment is a significant contributor to myocardial T1 relaxation time. Vasodilator stress T1 mapping may be a potential technique to assess coronary reserve without the need for contrast administration. Performing T1 mapping soon after vasodilator stress may affect ECV measurements given that hyperemia alone substantially alters T1 values.

## Funding

N/A.

